# Mechanistic insight into the oxidative degradation of monoclonal antibodies: relevance to developability and the design of stable pharmaceutical formulations

**DOI:** 10.1042/BST20250134

**Published:** 2026-05-14

**Authors:** Christian Schöneich

**Affiliations:** Department of Pharmaceutical Chemistry, The University of Kansas, 2093 Constant Avenue, Lawrence, KS 66047, U.S.A.

**Keywords:** antibody, developability, Oxidation, pharmaceutical formulation, Protein

## Abstract

Oxidation represents a key pathway for the chemical degradation of therapeutic monoclonal antibodies (mAbs), and chemical liabilities such as amino acid residue oxidation are an integral part of developability studies. Mechanistically, oxidation reactions in formulations of therapeutic mAbs are frequently not well understood, as critical information such as the nature of the oxidant(s) is frequently lacking. This brief review summarizes recent screening and developability studies of therapeutic proteins specifically focusing on oxidative liabilities and discusses these data in view of mechanistic and complementary analytical information on the oxidation of amino acid residues in specific protein sequences.

## Introduction

Chemical stability is one important parameter for the design of safe and efficacious formulations of therapeutic proteins. A major degradation pathway in these formulations is oxidation, though mechanistically this pathway is significantly less understood compared with other degradation routes such as, e.g., hydrolysis. There are various reasons for this, chief among them the facts that (i) the nature of oxidants in pharmaceutical formulations is frequently unknown, (ii) the nature of oxidants can change depending on the formulation composition, the identity and quantity of impurities, and the type of stresses the formulation is exposed to, and (iii) all amino acid residues of a protein are susceptible to oxidation, though preferential targets are the aromatic and sulfur-containing amino acids. Despite the complexity of reactions, it is possible to distinguish to some extent between individual oxidation pathways through careful analysis of mechanisms and reaction products. This entails the use of complementary analytical methods, including high-resolution mass spectrometry (MS) and nuclear magnetic resonance spectroscopy. Individual oxidants may be monitored by specific tests (e.g., the Amplex Red assay for hydrogen peroxide [1,2]) or, if free radicals are involved, by spin-trapping in combination with either electron spin resonance spectroscopy or mass spectrometry [3].

**Figure 1:**
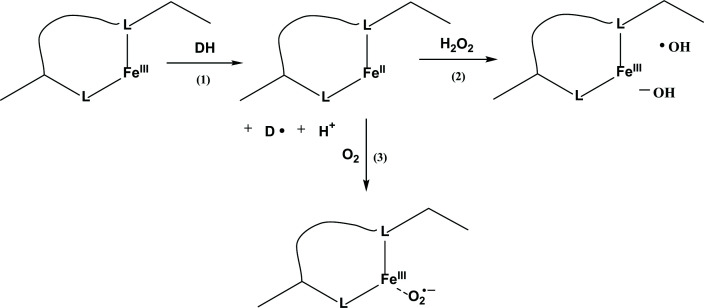
Schematic representation of site-specific reactions at a protein-bound FeIII (L represents metal-binding ligands). ^Schematic representation of site-specific reactions at a protein-bound FeIII (L represents metal-binding ligands).^

Protein oxidation reactions can be mediated by reactive oxygen species (ROS) generated in the bulk solution/environment and/or by site-specific processes. The latter confine the oxidation reactions(s) to select protein domains where specific factors such as amino acid composition, peptide sequence, and/or geometry allow for efficient oxidant generation and reactivity. Site-specific processes are significant for metal-catalyzed protein oxidation supported by the preferential binding of metals to select protein domains [4,5]. Critical to site-specific oxidation is the binding of a redox-active metal, which is in or can be converted to a reduced oxidation state (either through a reductant or light) and is available for reaction with either oxygen or peroxides within the metal-binding site (importantly, peroxides can function as reductants for redox-active metals [6]). This is schematically illustrated in Figure 1, where L denotes metal-binding ligands located on the protein.

Such conditions will be relevant for the case study on Fe^II^-dependent polyreactivity, presented below. Protein oxidation can also be mediated by light. Here, the nature and intensity of the incident light will play a major role with respect to the nature and yield of photo-oxidation products. The incident light may directly interact with chromophores of the protein but also with chromophores present through excipients and impurities, a situation that may lead to a variety of reactive intermediates originating from both protein and excipients [7]. Potential chromophores on the protein include tryptophan (Trp), as further discussed below, and protein degradation products (e.g., Trp oxidation products, age-related glycation end products, thiolato-cobalamine adducts, etc.) [7,8]. Potential chromophores on excipients include iron complexes of buffers such as citrate, His, and aspartate (Asp), where exposure to light can lead to the generation of a variety of ROS as well as a powerful reductant, the carbon dioxide radical anion (^•^CO_2_^−^) [9–14]. Formulations of therapeutic proteins will be exposed predominantly to visible and near UV light, and a longstanding question is how visible light exposure can trigger protein oxidation despite the fact that individual amino acids in proteins do not significantly absorb visible light. A potential answer to this question will be provided below.

This review will focus predominantly on monoclonal antibodies (mAbs). Typically, the excipients in commercial formulations of mAbs encompass combinations of buffers (predominantly histidine (His), phosphate, citrate, and/or acetate), cryo- or lyoprotectants (e.g., sucrose or trehalose), surfactants (e.g., polysorbate or poloxamer), amino acids (e.g., arginine (Arg), glycine (Gly), proline (Pro), or lysine (Lys)), and tonicity modifiers [15,16]. The pH values of monoclonal antibody formulations predominantly cover the range of pH 5–7 [15]. Typical excipient concentrations vary depending on whether the products are designed as liquid versus lyophilized or low- versus high-antibody-concentration formulations [15,16]. Especially the presence of surfactants at concentrations above the critical micelle concentration will create biphasic environments where oxidation reactions may proceed more efficiently within the lipid cores of surfactant micelles [17–19]. While proteins will not be in contact with these micellar lipid cores, oxidation processes within these cores can create ROS (e.g., lipid hydroperoxides), which may subsequently translocate to micellar surfaces (peroxidized lipids are more surface-active than non-peroxidized lipids and lower the interfacial tension of a hexadecane/water bilayer [20]). These ROS can react with the proteins at the micelle surface, in the bulk solution or at other interfaces present in the formulation (e.g., the air-water interface).

Mechanistic details could be of great assistance for the design of stable formulations through lead optimization, the interpretation of developability assessment, and mitigation strategies. Therefore, this brief review will focus on mechanistic aspects of important oxidation processes in pharmaceutical formulations and their impact on design, manufacturing, storage, and patient administration. Specific emphasis will be placed on the developability of therapeutic mAbs, mechanistic details on the oxidation of Trp and methionine (Met), and the oxidative degradation of antibody–drug conjugates (ADCs).

## Developability

Over the last years, much research activity has focused on the developability of therapeutic proteins, predominantly mAbs [21–23]. For antibodies, Zhang et al. [22] define that “developability refers to the likelihood that an antibody candidate will become a manufacturable, safe, and efficacious drug.” Among many other parameters, the identification of chemical modifications and/or chemical liabilities is critical at an early time point during discovery and development [21]. The amino acid residues Met and Trp are most frequently characterized as targets for oxidation [24], with potential consequences for specificity, affinity, or pharmacokinetics, depending on the location of these targets in a mAb (variable versus constant domain). However, depending on the stress conditions, other amino acid residues can become susceptible to oxidation, including tyrosine (Tyr), phenylalanine (Phe), His, cysteine (Cys) and cystine, and even the aliphatic amino acids (see below). Future studies employing analytical techniques that provide higher sensitivity and resolution may lead to an expansion of the list of potential oxidation targets and products in therapeutic proteins. This may become relevant for the mechanistic understanding of immunogenicity [25–28].

A recent case study describes the attempted correction of a potential oxidation liability, Trp^104^ in the CDR3 domain on the heavy chain (HC) of an IgG1 mAb (mAb B), through site-directed mutagenesis [21]. HC Trp^104^ was oxidized to an extent of 15%–18% during exposure to 200 Wh/m^2^ UV and 1200 kluxh visible light at 25°C, associated with >6% formation of higher molecular weight (HMW) species and the loss of affinity (the photo-sensitivity of HC Trp^104^ had also been reported by others [29]). For developability studies, 10 site-directed mutants of mAb B were constructed replacing Trp^104^ with Tyr, valine (Val), serine (Ser), alanine (Ala), threonine (Thr), leucine (Leu), Gly, asparagine (Asn), glutamine (Gln), or Phe. Only the Tyr^104^Phe mutant displayed a high binding affinity comparable to the wild-type (wt) protein while the other mutants exhibited up to four orders of magnitude increased dissociation constants during surface plasmon resonance experiments. However, the Trp^104^Phe mutant revealed a tendency for increased self-association and viscosity, ultimately leading to the decision to proceed with the uncorrected wt protein and to accept the potential liability of Trp^104^ oxidation. A closer look at the light stress applied to evaluate the photo-sensitivity of mAb B reveals that the light exposure was way above worst-case scenarios expected for a production facility (1.5–3.0 Wh/m^2^ UV and 43–260 kluxh visible light [30]) and indoor artificial lighting representative of a clinic environment (40 kluxh visible light [31]), respectively. The fact that visible light does induce protein photo-degradation [32–35] leads to the question of the underlying mechanism(s) (recently reviewed [7]). Results by Kim et al. [36] may provide a long-sought answer: the authors characterized charge-transfer (CT) complexes between Trp and O_2_ as a result of a tight interaction in specific protein folds, for which DFT calculations reveal optical absorptions around 445 and 480 nm. In a specific example, the CT excitation converts a complex between a singlet ground state of Trp (^1^Trp) of maltose-binding protein and triplet oxygen (^3^O_2_) to two nearly degenerate excited triplet states, in which Trp is present as radical cation and oxygen has accepted an electron to yield superoxide (O_2_^•−^) (reaction 4).

(4) $${}^{1}{\text{Trp-}}^{3}{\text{O}}_{2}\text{⟶}{\mathrm{Trp}}^{+•}{{\text{-O}}_{2}}^{•-}$$

Ultimately, these excited triplet complexes may lead to the generation of singlet oxygen (^1^O_2_) through spin flip and internal conversion processes [36,37]. Whether or not such protein folds, supporting CT complexes between Trp residues and oxygen, exist in mAbs remains to be shown. Machine learning studies revealed that solvent exposure and some secondary structure promoted Trp photo-oxidation most efficiently in proteins [38]. An increased solvent exposure appears counter-intuitive to a protein fold that promotes a tight interaction between Trp and oxygen; however, it is possible that a fraction of singlet oxygen generated in these protein folds escapes to react with additional solvent-exposed Trp residues. In proteins, ^1^O_2_ reacts most efficiently with the side chains of Trp, His, Tyr, Met, and Cys (with rate constants on the order of 10^7^-10^8^ M^−1^s^−1^) [39]. However, for a given amino acid such as Trp, the reactivity with ^1^O_2_ can vary significantly based on the nature of the protein, the solvent exposure, and the specific environment of Trp [40].

A susceptibility to oxidation is also a liability directly associated with the acquisition of polyreactivity, i.e. the ability of a mAb to bind more than one unrelated antigen [23,41]. Lecerf et al. demonstrated that the exposure of antibodies to 200 μM Fe^II^ can cause polyreactivity, which, by comparison of a library of 119 IgG1 mAbs, correlates with specific sequence features [41]. Most pronounced is a positive correlation with the number of Pro residues in the HC CDR2 domain, while less positive correlations exist with Thr on the HC CDR1 domain, positively charged amino acids on the HC framework region 1, and Asp on the HC framework region 2. For the light chain (LC), positive correlations are apparent with positively charged amino acids on the LC framework region 2, and Asp and isoleucine (Ile) on the LC framework region 1. Overall, more sequence features of the variable domain in the LC (V_L_) showed a positive correlation with Fe^II^-dependent polyreactivity. In general, polyreactivity is connected to the structural dynamics of the antigen-binding site where greater flexibility may lead to better adaptability towards various antigens [42]. Polyreactivity has been associated with various features, including elevated net charges, surface patches of positively charged or hydrophobic amino acids [42–44], but also with the presence of negatively charged amino acids [42]. The latter is inconsistent with the *in silico* predictions by Harvey et al. [44]. Specifically, with regard to the results of Lecerf et al., a mechanistic interpretation needs to look at the various roles that Fe^II^ can play in the formulations of mAbs and how these depend on the protein and the nature of the excipients. Lecerf et al. consider that Fe^II^ binding to mAbs may affect conformation and/or flexibility but ultimately conclude that Fe^II^ acts as a pro-oxidative agent [41], implying a role as a promotor/catalyst for redox reactions. Other studies did not detect any significant effect of Fe^III^ binding to a mAb on secondary and tertiary structure [45]. However, depending on the nature of the mAb or mAb domain, Fe^III^ binding does [45] or does not [46] affect thermal stability.

The presence of Fe^II^-complexing ligand geometries will define the locations of Fe^II^-binding to the protein. If the protein were the only ligand present in the formulation, Fe^II^ would naturally associate with the protein. In these protein/Fe^II^ complexes, L_x_Fe^II^ can react with O_2_, generating L_x_Fe^III^ and O_2_^•−^ (Figure 1, reaction 3) or with other oxidants such as H_2_O_2_ or organic peroxides (Figure 1, reaction 2) (L_x_ denotes the ligand sphere around the metal). The reaction of L_x_Fe^II^ with H_2_O_2_ is generally referred to as the Fenton reaction, yielding either hydroxyl radicals (HO^•^), ferryl species ([L_x_Fe^IV^ = O]^n+^), and even ligand radicals (L^•^), depending on pH and nature of the ligands [47–50]. Both HO^•^ and [L_x_Fe^IV^ = O]^n+^ are strong oxidants [50] and can react site-specifically with amino acid residues located within or near the metal-binding site (while the ligand radical L^•^ would be generated on the protein if L is the protein ligand). In contrast, the O_2_^•−^ radical anion will not efficiently react with the protein and rather dismutate to H_2_O_2_, which would then be available for the Fenton reaction. Hence, an experiment where a mAb would be exposed to Fe^II^ and H_2_O_2_ should be able to reveal amino acids that are involved in metal-binding and/or are located near the metal-binding site. Such an experiment of hydroxyl radical footprinting was provided by Glover et al., where 3 mg/ml trastuzumab was exposed to 50 μM Fe^II^ and 1 mM H_2_O_2_ in 10 mM sodium acetate buffer, pH 5.5, containing 60 mM sucrose and 0.02 mg/ml polysorbate 20, followed by peptide mapping and analysis by high-performance liquid chromatography-MS [51]. In these peptide maps the authors differentiated between oxidation and carbonylation products through the design of their search parameters for products in the MS data [51,52] (however, it is understood that both types of products are generated through oxidation). Carbonylation products are generated mainly from the amino acid residues Pro, Lys, Arg, and Thr [5], which is significant with regard to Pro, the amino acid residue showing the strongest correlation with Fe^II^-dependent polyreactivity [41]. Oxidation products were detected predominantly on the tryptic peptide HC sequences Trp^99^–Lys^124^ (CDR3 domain), Asp^151^-Lys^213^, Asp^252^-Arg^258^, Glu^359^-Lys^363^, and Trp^420^-Lys^442^ (for a trastuzumab sequence, see Kyoto Encyclopedia of Genes and Genomes, KEGG; KEGG Drug database; https://www.kegg.jp/entry/D03257). Significantly less oxidation was detected for the LC, mainly in the sequence Ala^25^-Lys^42^. Carbonylation was observed predominantly in the HC sequences Tyr^60^-Gly^66^ and Asp^252^-Arg^258^ (unfortunately no product information is available for the sequence Val^218^-Lys^225^, which includes Pro^220^, but also an additional tryptic cleavage site, Lys^221^; this additional tryptic cleavage site can result in the formation of small hydrophilic peptides, which may not be resolved during chromatographic separation). One potential reason for the lower susceptibility of the LC may be the generally lower number of targets for carbonylation (Pro, Lys, Arg, Thr) in the LC as compared with the HC (https://www.kegg.jp/entry/D03257). In addition, the HC contains a significantly higher number of general oxidation targets (His, Tyr, Met, Trp) (https://www.kegg.jp/entry/D03257).

An important detail is that neither significant yields of oxidation nor carbonylation products were detected by Glover et al. in the HC CDR2 domain of trastuzumab as a result of exposure to Fe^II^ and H_2_O_2_ [51]. In contrast, Lecerf et al. identified a strong positive correlation between Fe^II^-dependent polyreactivity with Pro in the HC CDR2 domain by comparison of 119 IgG1 mAbs [41]. Significantly less oxidation and negligible carbonylation were detected on the trastuzumab LC, contrasting the finding that Fe^II^-dependent polyreactivity correlated with more sequence features on the LC. Of additional note is that (i) trastuzumab represents a mAb that shows high sensitivity towards Fe^II^-dependent polyreactivity [41], and (ii) polyreactivity of trastuzumab was also induced by the exposure to an unrelated oxidant, hypochlorite (OCl^−^) [41]. Importantly, OCl^−^ shows highest reactivity towards sulfur-containing protein amino acid residues (Met, Cys), with rate constants on the order of 10^7^-10^8^ M^−1^s^−1^ at physiologic pH [53,54]. This compares to several orders of magnitude lower rate constants for the reaction of OCl^−^ with Trp (1.1 × 10^4^ M^−1^s^−1^), Lys (5.0 × 10^3^ M^−1^s^−1^), Tyr (44 M^−1^s^−1^), Arg (26 M^−1^s^−1^), and Gln/Asn residues (0.03 M^−1^s^−1^) [53].

These results lead to multiple possible interpretations. The positive correlation of Fe^II^-dependent polyreactivity with the frequency of Pro residues in the HC CDR2 domain may not be related to an oxidative modification of Pro but rather to a conformational role. In order to shed light on this question, Lecerf et al. exposed trastuzumab to OCl^−^ [41]. However, OCl^−^ does not react efficiently with simple aliphatic amino acid residues but rather targets Met [53]; in fact, the exposure of trastuzumab to Fe^II^ and H_2_O_2_ predominantly targets peptide sequences that contain Met [51]. In contrast, there is no particular correlation with the frequency of Met on the CDRs of IgG1 mAbs and Fe^II^-induced polyreactivity [41]; a slight positive correlation is observed with the frequency of Met in the LC framework 1 [41].

Compared with Pro on the HC CDR2 domain, a less but still significant positive correlation of Fe^II^-dependent polyreactivity was observed with the frequency of Thr on the HC CDR1 domain [41]. Glover et al. did not observe Fe^II^/H_2_O_2_-dependent oxidation on the peptide sequence Asp^31^-Arg^38^ of trastuzumab, assigned to the CDR1 domain. However, Yang et al. exposed an unspecified IgG1 to Fe^II^/H_2_O_2_, albeit at higher concentrations (2 mM each) compared with trastuzumab by Glover et al. and reported that among all residues converted to carbonyl products (Arg, Lys, Thr, Pro), HC Thr^28^ exhibited the highest yields [55]. It is not clear whether Thr^28^ is located within or in close vicinity to the CDR1 domain, but in both instances the chemical modification of this Thr residue may modify antigen binding, as it has been reported that framework residues can affect CDR conformation and binding affinity [56].

Fe^II^-dependent polyreactivity displays positive correlations with positively charged amino acids on the LC framework region 2, and Asp and isoleucine (Ile) on the LC framework region 1. Relatively low yields of protein oxidation, but no carbonylation, were detected by Glover et al. [51] on the trastuzumab LC sequence Ala^25^-Lys^42^, while also Yang et al. [55] did not document any carbonyl formation in this section of the unspecified IgG1. Notably, the peptide sequence Ala^25^-Lys^42^ contains the aromatic amino acid residues Trp and Tyr (KEGG Drug database; https://www.kegg.jp/entry/D03257), but no positive correlation was reported for these residues with Fe^II^-dependent polyreactivity [41].

Overall, except possibly for Thr^28^, there is to date little experimental evidence for a direct oxidation of the specific amino acid residues, which have been identified to correlate most positively with Fe^II^-dependent polyreactivity. On the other hand, the exposure of trastuzumab to Fe^II^ and H_2_O_2_ leads to significant oxidation of Met, an amino acid that does not correlate with Fe^II^-induced polyreactivity.

The reaction conditions of Lecerf et al. are sufficient for the Fe^II^-dependent generation of oxidants within the time frame of the experiment: polyreactivity was monitored after a 10 min exposure of mAbs to 200 μM FeSO_4_ in phosphate-buffered saline [41], which contains about 10 mM phosphate buffer at pH ∼7.4 [57]. Phosphate and increasing pH promote the oxidation of Fe^II^ by O_2_ [58], and based on data from Mao et al. [58], an estimate of the pseudo-first-order rate constant for Fe^II^ oxidation to Fe^III^ in air-saturated aqueous solution, containing 10 mM phosphate at pH 7.4, yields k_obs_ ≈ 5.6 × 10^−3^ s^−1^, translating into a half-life on the order of 2 min. We note that the oxidation kinetics in the experiments of Lecerf et al. [41] will initially be closer to second order, as 200 μM FeSO_4_ reacts with about 250 μM O_2_ (air-saturated aqueous solution), for which a first half-life of about 3 min. can be estimated. This reaction will produce 0.5 mol H_2_O_2_ per mol Fe^III^, ultimately generating a total of 100 μM H_2_O_2_. Therefore, we expect that the reaction conditions of Lecerf et al. generate about 50 μM H_2_O_2_ within the first 3 min of the experiment. During the course of reaction, H_2_O_2_ can react with unreacted Fe^II^ to yield HO^•^ and/or [L_x_Fe^IV^ = O]^n+^. The rate constant for this process is expected to be on the order of 5 × 10^3^ M^−1^s^−1^ [59,60] for Fe^II^ complexed by oxygen ligands of the protein [61] (by analogy to the rate constant for the reaction of ferrous citrate with H_2_O_2_ [59,60]). Hence, it can be estimated that the ca. 50 μM H_2_O_2_ generated within the first 3 min of the experiment will convert into either HO^•^ and/or [L_x_Fe^IV^ = O]^n+^ with a first half-life of <7 s. These species are likely responsible for a significant fraction of the carbonyl yield [5] (the reaction of HO^•^ with sulfides such as present in Met does not efficiently yield sulfoxide [62]).

Alternatively, H_2_O_2_ can react with the product Fe^III^ (equilibrium 5; k_5_ = 69 M^−1^s^−1^, k_−5_ = 0.11 s^−1^), generating the relatively strong oxidant L_x_Fe^III^(^−^OOH) [48].

(5) LxFeIII+H2O2⇌LxFeIII(OOH-)+H+

The latter species may well be responsible for a significant fraction of Met oxidation to Met sulfoxide reported by Glover et al. [51]. Notably, the concentrations of Fe^II^ applied by Lecerf et al. [41], Glover et al. [51], and Yang et al. [55] are not identical and are all significantly higher than iron concentrations that may be expected in pharmaceutical formulations (for a comparison of experimental conditions, see Table 1). For example, Bensaid et al. [63] quantified approximately 20 ppb iron (equivalent to 0.36 μM) in a formulation batch containing 20 mg/ml IgG1, 20 mM His, pH 6.0, 10% w/v sucrose, and 200 ppm polysorbate 80, originating from drug substance manufacturing. Majumder et al. detected ca. 182 ppb iron (equivalent to ca. 3.3 μM) in a drug substance batch of an IgG1 [64], while iron concentrations as high as 1-9 μM were detected in some other batches of mAbs [65,66].

**Table 1:** Comparison of the oxidizing conditions in the relevant references

Reference	mAb	Other excipients	Buffer, pH	[Fe^II^]	Trigger	Potential oxidants^a^
Lecerf et al. [41]	IgG1		PBS^b^	200 μM	O_2_ (air)	HO^•^, [L_x_Fe^IV^ = O]^n+^, L_x_Fe^III^(^−^OOH)
Glover et al. [51]	IgG1	60 mM sucrose, 0.02 mg/ml PS20^c^	10 mM NaAc^d^, pH 5.5	50 μM	1.0 mM H_2_O_2_	HO^•^, [L_x_Fe^IV^ = O]^n+^, L_x_Fe^III^(^−^OOH)
	IgG1	60 mM sucrose, 0.02 mg/ml PS20	10 mM NaAc, pH 5.5	100 μM	0.1 mM H_2_O_2_	
	IgG1	60 mM sucrose, 0.02 mg/ml PS20	10 mM NaAc, pH 5.5	100 μM	1.0 mM H_2_O_2_	
Yang et al. [55]	IgG1	Unknown	50 mM Na succinate, pH 6.5	2.0 mM	2.0 mM H_2_O_2_	HO^•^, [L_x_Fe^IV^ = O]^n+^, L_x_Fe^III^(^−^OOH)

a Generated through the reaction of H_2_O_2_ with either Fe^II^ or Fe^III^.

b PBS = phosphate buffered saline (pH ∼7.4).

c PS20 = polysorbate 20.

d NaAc = sodium acetate.

Glover et al. [51] inferred Fe^II^ binding to an IgG1 as a rationale for the site-specific oxidation induced by H_2_O_2_, consistent with the mechanism of Stadtman [4]. The binding of Fe^III^ to IgG1 is also critical for the site-specific oxidation of Thr^259^ induced by light [67,68], where further characterization of the Fe^III^ binding site was provided by molecular modeling experiments [69]. Importantly, the specific Fe^III^ binding site critical for light-induced degradation overlaps with one of the sites identified by Glover et al. [51] in their site-specific oxidation experiments induced by Fe^II^/H_2_O_2_. Isothermal titration calorimetry further confirmed the binding of Fe^III^ to a mAb (IgG2), indicating the binding of up to 20 ± 1 Fe^III^ with an overall K_D_ ≤ 0.18 mM [45].

## Methionine

**Figure 2:**
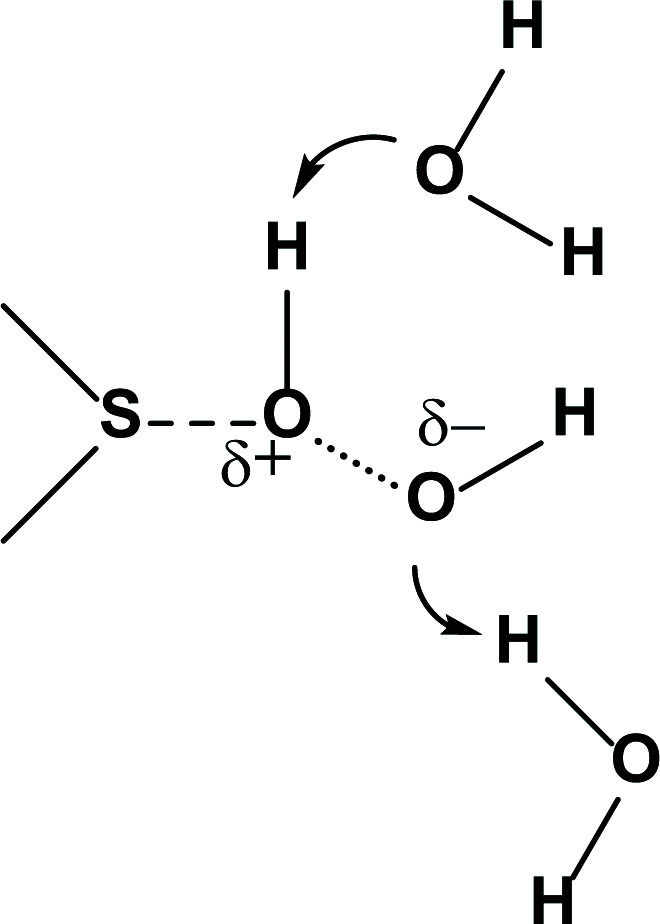
The role of hydrogen bonding in charge separation along the O-O bond of H2O2 during sulfide oxidation. The role of hydrogen bonding in charge separation along the O–O bond of H_2_O_2_ during sulfide oxidation.

Machine learning algorithms were applied to predict Met oxidation (to Met sulfoxide) based on the analysis of large datasets derived from various sources, including *in vivo* protein oxidation and the oxidation of therapeutic proteins [38,70,71]. In general, oxidation is favored when the Met residue is present in a domain with higher solvent-accessible surface area (SASA) and higher flexibility, and when the Met residue experiences fewer contacts with neighboring residues; in addition, the distance to nearby Met and aromatic residues plays a role, where the interaction of Met with aromatic acids can protect Met from oxidation when the distance between the Met sulfur atom and the center of mass of the aromatic ring is within 7 Å [70,72]. An exact comparison between different machine learning studies can be complicated by the fact that they frequently used different oxidants for the experimental generation of Met sulfoxide. An important parameter controlling protein Met oxidation by H_2_O_2_ is the 2-shell water coordination number (2SWCN) [73], essentially quantifying how many water molecules are present within a specific radius around the sulfur atom of Met, available for hydrogen bonding to H_2_O_2_. Such hydrogen bonding assists in charge separation within H_2_O_2_, which represents the rate-determining step in the oxidation of sulfides by H_2_O_2_ (Figure 2).

The correlation of Met oxidation with 2SWCN is no contradiction to the importance of the SASA, identified by machine learning, as a higher SASA will likely also result in a higher 2SWCN. However, Tavella et al. reported that hydrogen bonding with additional hydroxyl groups present on amino acid residues, such as Ser, Thr, and Tyr, must be considered for an even better correlation with experimentally observed Met oxidation [74]. Therefore, these authors introduced a new parameter, WCN-OH, which takes into account both water molecules and hydroxyl groups on amino acid side chains as hydrogen bonding partners. The WCN-OH provided a better positive correlation, predicting all oxidation-susceptible Met residue in a series of antibodies [74].

In alignment with the predictions by machine learning, a solvent-accessible Met residue within the CDR of a mAb, HC-Met^111^, was significantly more susceptible to oxidation compared with a buried Met residue located within the same CDR, HC-Met^115^ [61]. However, the buried HC-Met^115^ exhibited stereoselectivity in its reaction with H_2_O_2_, generating specific Met sulfoxide diastereomers: in the absence of a denaturant, H_2_O_2_ generated the R-sulfoxide, while the presence of a denaturant such as urea favored formation of the S-sulfoxide [61]. While the side chain orientation of the S-sulfoxide was similar compared with that of non-oxidized Met^115^, the R-sulfoxide led to rotation along the C_β_-C_γ_ bond, leading to weakening of peptide backbone hydrogen bonding between HC-Asp^116^ and HC-Arg^106^. As a result, deuterium uptake during hydrogen-deuterium exchange experiments was faster for the R-sulfoxide as compared with the S-sulfoxide and non-oxidized protein. However, these conformational changes had no impact on antigen binding, indicating that oxidation of HC-Met^111^ and HC-Met^115^ in the CDR was benign for this specific mAb [61].

Met residues are prone to photo-oxidation under conditions recommended by the International Council for Harmonization (ICH) Q1B guideline, i.e. exposure to cool white light at 1.2 million luxh and UV light at 200 Wh/m^2^ [38]. It is likely that under these conditions the UV component triggers type II photochemistry of Trp, generating singlet oxygen (^1^O_2_). ADCs containing photosensitive payloads would be specifically susceptible to this type of photodegradation. In fact, an IgG1 conjugated to a topoisomerase I inhibitor, a camptothecin derivative, revealed significant oxidation of three Met residues, Met^257^, Met^433^, and Met^363^, even under exposure to ambient light [75]. In contrast, when the camptothecin derivative was spiked into an IgG1-containing solution (without direct conjugation), significantly less Met oxidation was observed. This trend correlated with the formation of higher molecular weight species (HMWS) [75] and a similar trend was earlier observed for a model ADC containing eosin [76]. In contrast, Thiess et al. observed that comparable yields of HMWS were generated for a camptothecin derivative-containing ADC and a mAb solution into which the campothecin derivative was spiked [77]. The reasons for these differences are presently unclear but it should be noted that the different studies used ADCs with different drug-antibody ratios (DARs) and different types of camptothecins.

## Perspectives

- A detailed understanding of the mechanisms of therapeutic protein oxidation in pharmaceutical formulations would be important for the interpretation of machine learning and developability studies designed to predict oxidation sites and yields in new protein candidates based on the analysis of large existing data sets.
- Currently, Met and Trp are considered key oxidation sites in therapeutic proteins but additional targeted studies on the oxidation of existing and future therapeutic proteins under formulation conditions may lead to an expansion of the list of potential oxidation targets. Research in this area should also focus on Cys oxidation, as low levels of Cys may be present in certain mAbs due to incomplete disulfide bond formation.
- High sensitivity and resolution analytical methods should be employed for a thorough characterization of protein oxidation products and focus not only on the most susceptible targets. The identification of additional oxidation products may become relevant for the understanding of immunogenicity.

## Open Access

Open access for this article is provided via our Subscribe to Open programme, made possible through the support of our subscribing institutions. Learn more here: https://portlandpress.com/pages/open_access_options_and_prices#conditional.

## Abbreviations

Ala: alanine
ADCs: antibody–drug conjugates
Asn: asparagine
Asp: aspartate
CT: charge-transfer
Cys: cysteine
Gln: glutamine
Gly: glycine
HC: heavy chain
HMW: higher molecular weight
HMWS: higher molecular weight species
Leu: leucine
LC: light chain
Lys: lysine
MS: mass spectrometry
Met: methionine
mAbs: monoclonal antibodies
Phe: phenylalanine
Pro: proline
ROS: reactive oxygen species
Ser: serine
SASA: solvent-accessible surface area
Thr: threonine
Trp: tryptophan
Val: valine
wt: wild-type
2SWCN: 2-shell water coordination number
